# Research on Identification Method of Wear Degradation of External Gear Pump Based on Flow Field Analysis

**DOI:** 10.3390/s20144058

**Published:** 2020-07-21

**Authors:** Rui Guo, Yongtao Li, Yue Shi, Hucheng Li, Jingyi Zhao, Dianrong Gao

**Affiliations:** 1 Hebei Provincial Key Laboratory of Heavy Machinery Fluid Power Transmission and Control, Yanshan University, Qinhuangdao 066004, China; liyongtao@stumail.ysu.edu.cn (Y.L.); zjy@ysu.edu.cn (J.Z.); gaodr@ysu.edu.cn (D.G.); 2 State Key Laboratory of Fluid Power and Mechatronic Systems, Zhejiang University, Hangzhou 310027, China; 3 Key Laboratory of Advanced Forging & Stamping Technology and Science, Yanshan University, Qinhuangdao 066004, China; sy100517@stumail.ysu.edu.cn (Y.S.); andylhc@stumail.ysu.edu.cn (H.L.); 4 Hebei Key Laboratory of Special Delivery Equipment Yanshan University, Qinhuangdao 066004, China

**Keywords:** external gear pump, degradation state recognition, numerical simulation, wearing clearance

## Abstract

As a kind of hydraulic power component, the external gear pump determines the performance of the entire hydraulic system. The degradation state of gear pumps can be monitored by sensors. Based on the accelerated life test (ALT), this paper proposes a method to identify the wear degradation state of external gear pumps based on flow field analysis. Firstly, the external gear pump is theoretically analyzed. Secondly, dynamic grid technology is used to simulate the internal flow field of the gear pump in detail. Finally, the theoretical and simulation results are verified by the ALT. The results show that this method can effectively identify the wear degradation status of four sample pumps. The results of the work not only provide a solution to the research on the wear degradation of external gear pumps, but also provide strong technical support for the predictive maintenance of hydraulic pumps.

## 1. Introduction

The gear pump is one of the most typical power components of hydraulic systems, which has the advantages of high working efficiency and structural simplicity. Generally, the gear pump is subjected to the effects of various environmental factors such as wear, temperature, load and others that can easily lead to the degradation of a pump and have a serious impact on the safe operation of the whole system [1].

Thus, it is essential to evaluate the degradation state of a gear pump for its structural improvement and condition monitoring. In recent years, several studies identifying the degradation state of gear pumps have been published. Li et al. [2] proposed a method to extract the degradation characteristics of the hydraulic pump based on the relative entropy, and predicted the remaining life of the plunger pump. Ma et al. [3] regarded the degradation mechanism and process as the criteria to evaluate the reliability, then provided an analytical method to the performance reliability of the plunger pump. Tian et al. [4] presented an identification method based on improved multi-fractal detrended fluctuation analysis and semi-supervised Mahalanobis fuzzy c-means, then verified its effectiveness with data available from experiments. According to the diagnosis features extracted from the vibration signal spectrum of the oil extraction pump, Wang et al. [5] processed the spectrum of different fault states by using the fuzzy membership function, realizing the correct classification and condition recognition of different pump fault spectra. Wang et al. [6] proposed spatial information entropy as a new degradation feature of pump based on the research of permutation entropy algorithm, and used a fuzzy c-means algorithm to diagnose the degradation state of piston pump.

These aforementioned studies are all related to the data obtained from experiments. However, optimizing the performance and structure of gear pumps by experimental studies can cause high energy consumption and costs that will greatly consume manpower and material resources. Therefore, a method with simulation analysis has been investigated by scholars to conduct research on gear pumps. Frosina et al. [7] studied the pressure in the cavity of the driving gear and the driven gear through the combination of virtual simulation for gear pump and test. Campo et al. [8] verified the numerical analysis of the gear pump with the experimental data, and identified the influence of the inlet and outlet pressure on the volumetric efficiency accessing an image velocity measurement method. Yoon et al. [9] proposed a simulation method of pump performance characteristic parameters, and the working process of the gear pump under high-speed extreme conditions is simulated by the immersed solid method. Castilla et al. [10] demonstrated the fluid flow inside the external gear pump with a complete 3D model and proved that the result is more accurate than 2D simulation. Močilan et al. [11] designed the gear pump with the parameterization function of ANSYS Gambit, analyzed the assembly by finite volume method, and proposed a model for predicting the dynamic behavior of the external gear pump.

**Figure 1:**
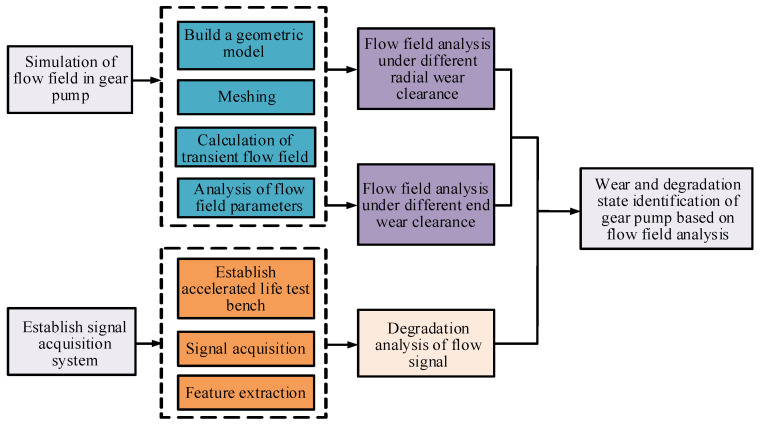
Flow chart of technical route.

From the above, it can be found that the research on gear pump by simulation technology mostly focuses on the general characteristics, and has not been involved in the aspect of wear degradation. In this paper, an identification method of wear degradation of an external gear pump is proposed based on flow-field analysis. With flow-field analysis of a gear pump, the simulation models under different wear clearance are constructed. FLUENT software is used to calculate the instantaneous characteristic data in different wear degree, by which to obtain the wear degradation characteristic index of gear pump. Finally, a performance degradation experiment of an external gear pump is established and the effectiveness of the method is verified by the parameters measured from experiment. The technical route of this paper is given in Figure 1.

## 2. Research on Wear Degradation Mechanism

Generally, the wear parts of gear pump mainly include gear end face and floating side plate, tooth top and shell cavity and tooth surface of gear. In this section, the relationship between wear and leakage of the gear pump is introduced.

### 2.1. Analysis of End Face Wear and Leakage

When the gear pump is working, there will be a layer of oil film between the floating bush sleeve and the gear end face, and a good oil film can reduce the wear rate. Because of the action of the radial unbalanced force, the thickness of the oil film will change. When the working pressure of the system increases, the overturning torque generated by the pressure difference in the working chamber enlarges. Then, the floating shaft sleeve is tilted, which disturbs the periodic change of the oil film and causes the rupture of oil film. In this condition, the end face between the floating bush and the gear are forced to contact with each other and the occurrence of wear is aggravated. In addition, the contaminant particles in the hydraulic oil are another reason for the aggravation of the wear between the gear end face and the floating side plate.

**Figure 2:**
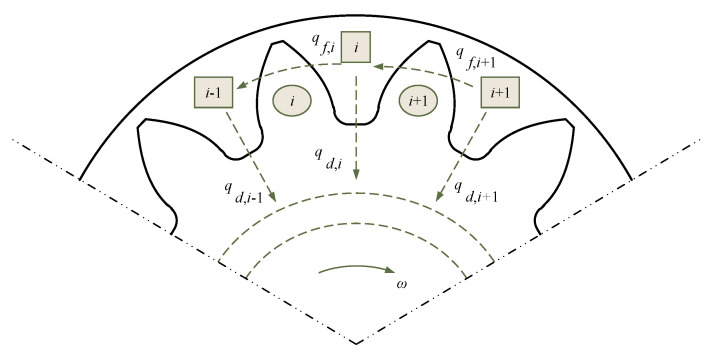
Schematic diagram of leakage path of gear pump end face.

The reduction of the output flow is the most direct result of the end face leakage. Figure 2 shows the leakage path of gear pump end face. The leakage paths described in Figure 2 are the leakages from the high-pressure chamber to the low pressure chamber and the leakage from the tooth cavity to the shaft center then to the low-pressure cavity from the clearance.

According to [12], the leakage flow at the end face of gear pump is calculated by the following equation:

(1) $$\Delta q_{s}=\frac{s^{3}\Delta P^{2}}{3\pi \mu }\sum_{n=1}^{\infty }\frac{1-{\left(\frac{R_{z}}{R_{e}}\right)}^{2n}}{1+{\left(\frac{R_{z}}{R_{e}}\right)}^{2n}}\left[\frac{\sin n\left(\theta_{1}-\pi \right)}{\theta_{1}}+\frac{\sin n\left(\theta_{0}-\pi \right)}{\pi -\theta_{0}}\right]\times \frac{1+\cos n\pi }{n^{2}}$$

where, *s* is the end-face clearance; *R_z_* is the radius of gear shaft; *R_e_* is the radius of tooth top circle; *θ*_1_ is the chord angle of pitch circle tooth thickness; and *θ*_0_ is the wrap angle of high pressure zone.

As already demonstrated in Equation (1), the total leakage of the end face is directly proportional to the third power of s, that is, the gap has a great influence on the leakage, which means its size must be strictly controlled.

### 2.2. Analysis of Radial Wear and Leakage

The radial wear of a gear pump is actually the wear between gear tooth tip and casing. In a similar principle to end face wear, the increase of the system pressure induces the increment of the radial force on the gear. Then, the gear shaft is deformed, contributing to the ruptures of oil film between the gear tooth tip and casing.

**Figure 3:**
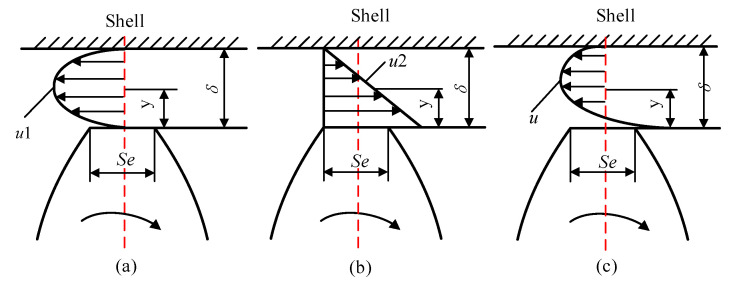
Gear pump radial leakage: (**a**) differential pressure flow; (**b**) shear flow; (**c**) synthetic flow.

Radial wear is the main cause of radial leakage increase which will also increase with system pressure. The reasonable design of the radial clearance has a very important influence on the life of the gear pump. An extra large design size will cause leakage increase, an extra small design size will lead to increased wear further expand the gap and increase the leakage. The leakage path of the radial gap of the gear pump is shown in Figure 3.

According to [12], the radial leakage Δ*q_δ_* of the gear pump is expressed by the following equation:

(2) $$\Delta q_{\delta }=B\left(\frac{\Delta P}{6\mu z_{0}S_{e}}\delta^{3}-\frac{\pi nR_{e}}{30}\delta \right)\times 60\times 10^{3}$$

where, *B* is the tooth width; Δ*P* is the pressure difference; *S_e_* is the thickness of the tooth tip; *δ* is the radial clearance; *Z*_0_ is the number of transition teeth; *μ* is the dynamic viscosity; *n* is the speed.

It can be conducted from the Equation (2) that only when *δ* = 0, can there be no leakage in the radial gap, which is impossible in actual design. Therefore, the leakage of the gear pump will definitely increase with the increase of the radial wear.

## 3. Flow-Field Simulation Analysis of Gear Pump

### 3.1. Introduction of Simulation Software

Computational fluid dynamics (CFD) is a method to intuitively describe the state distribution of the internal fluid field of the structure. In this paper, ICEM (Integrated Computer-Aided Engineering and Manufacturing) and FLUENT are selected as preprocessor and solver. The dynamic mesh model is used to simulate the internal flow field in the actual moving state. Its boundary condition can be defined by User Defined Functions (UDF). The moving grid is calculated by Equation (3).

(3) $$\frac{d}{dt}\int_{V}\rho \phi dV+\int_{\partial V}\rho \phi \left(\bar{u}-\bar{u_{s}}\right)d\bar{A}=\int_{\partial V}\Gamma \nabla \phi d\bar{A}+\int_{V}S_{\phi }dV$$

where, $\bar{u}$ is the velocity vector of the liquid; ${\bar{u}}_{s}$ is the deformation velocity of the moving mesh; ∂_*V*_ is the boundary of the control body *V*; Γ is the diffusion coefficient; *S_φ_* is the source term of the flux *φ*.

The theoretical displacement of the gear pump selected in this paper is 4 mL/r, the motor speed is 1470 rpm. The diameter of the oil inlet and outlet is 14 mm and 10 mm, respectively. According to [13], the problems of clearance fit and wear are considered. Thus, determine the initial radial clearance value *S_i_* = 20 μm and the end clearance value *δ_i_* = 50 μm.

**Figure 4:**
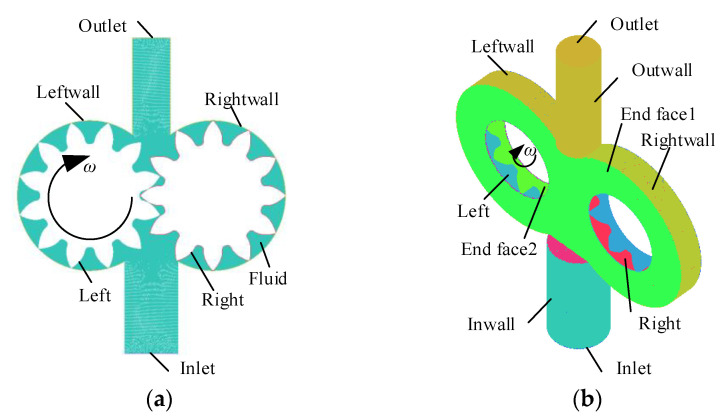
Mesh generation of gear pump simulation model: (**a**) 2D initial model; (**b**) 3D initial model.

Due to the symmetrical structure of the gear pump, a series of simplifications were made to the flow field model. A 2D model was used to simulate radial gap wear, and a 3D model was used to simulate end face gap wear [14,15]. The flow field initial mesh generation models of the gear pump are shown in Figure 4.

**Figure 5:**
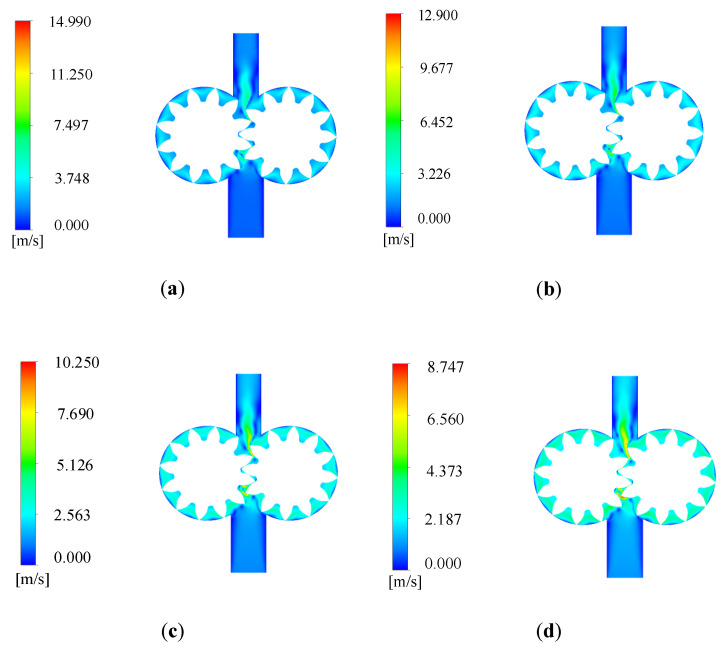
Velocity nephogram of 2D simulation model: (**a**) velocity nephogram at 0.01 s; (**b**) velocity nephogram at 0.02 s; (**c**) velocity nephogram at 0.03 s; (**d**) velocity nephogram at 0.04 s.

After the simulation, the velocity nephogram of the 2D simulation model shown in Figure 5 is obtained. It can be seen that the flow velocity of the oil changes uniformly with the rotation of the gear, and the flow speed of the oil is relatively large at the meshing and outlet of the gear, maximum liquid flow speed reaches 15 m/s. The change trend is consistent with the actual situation, which provides a basis for the simulation analysis of wear degradation [16].

**Figure 6:**
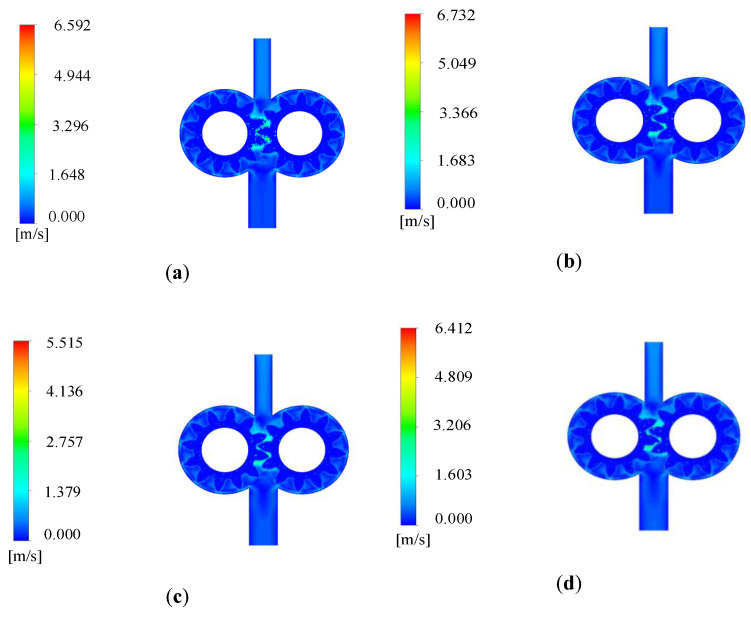
Velocity nephogram of 3D simulation model with 50% end face clearance: (**a**) velocity nephogram at 0.01 s; (**b**) velocity nephogram at 0.02 s; (**c**) velocity nephogram at 0.03 s; (**d**) velocity nephogram at 0.04 s.

The velocity nephogram of 3D simulation model with 50% end face clearance is shown in Figure 6. Compared with the velocity nephogram of 2D simulation model, the same characteristics can be observed. The flow velocity of the oil changes regularly with the rotation of the gear, and the flow velocity of the oil is larger at the meshing and outlet of the gear, with the maximum reaching 6.732 m/s.

According to the above analysis, it can be found that the simulation results of the internal flow field of the gear pump have a high similarity with the actual situation, which provides strong support for the correctness of the simulation model.

### 3.2. Theoretical Validation of Simulation Model

**Figure 7:**
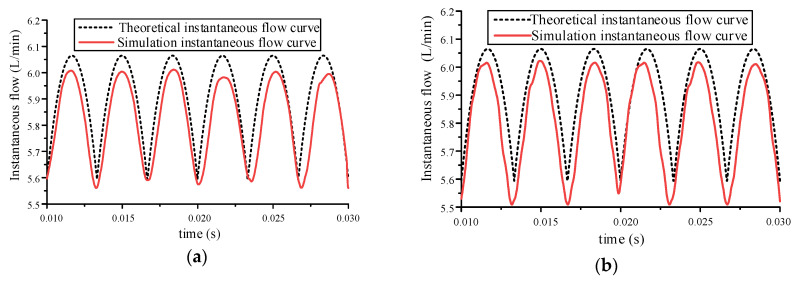
Comparison diagram of simulated instantaneous flow rate and theoretical instantaneous flow rate of gear pump outlet: (**a**) 2D model validation; (**b**) 3D model validation.

The most effective method to verify the correctness of the simulation model is to compare the instantaneous flow rate obtained by simulation with the flow rate calculated by theory. In this section, the gear pump speed is set to 1470 r/min and the operating condition is no-load. The simulation data of the outlet flow velocity is obtained through the establishment of the monitoring surface [17]. The comparison results of the instantaneous flow of the simulation model and the theoretical model are shown in Figure 7.

It can be seen from Figure 7 that although there are some errors in the instantaneous flow of the two models, their change trends are consistent. The reasons for these errors are as follows:

- In the simulation process, only instantaneous flow can be collected, because the acquisition time is very short, it will cause errors.
- The simplified model is used in the simulation, which is different from the actual model, so it will cause errors.
- The theoretical flow rate is the flow rate in an ideal state, and there are some differences from the actual flow rate.

The comparison results can be illustrated in the form of table. The comparison results of average flow of simulation model and theoretical model are shown in Table 1.

It can be seen from Table 1 that there is no significant difference between the average flow of the theoretical flow rate and the simulated flow rate, and the volume efficiency can be calculated as 99.7% and 99.5%, respectively. From the above analysis, it can be seen that the simulation model is accurate and effective.

### 3.3. Degradation Analysis of Simulated Flow Signal

**Figure 8:**
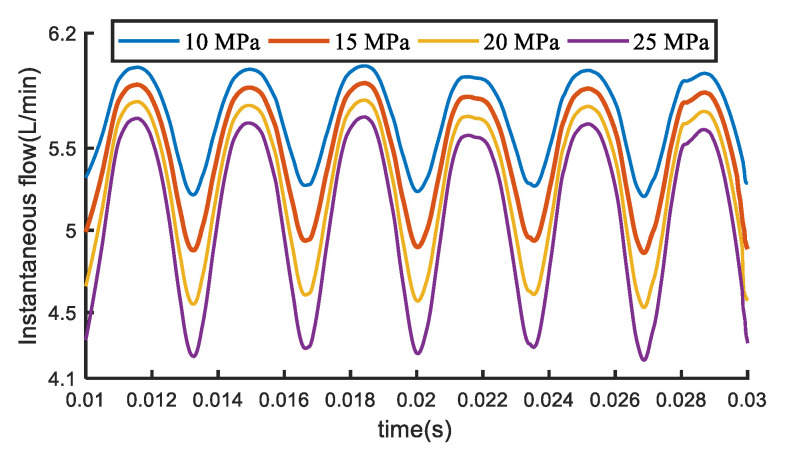
Contrast curves of simulated instantaneous flow of gear pumps under different pressure differences.

The flow field simulation in the gear pump under various pressures is discussed by using the 2D model [18]. Figure 8 compares the simulated instantaneous flow rate of the gear pump under different pressures. Because there are some discrete phenomena in the initial stage of the iteration when calculating, the flow contrast curve is intercepted from 0.01 s.

As can be seen from Figure 8, the instantaneous flow rate changes with time, showing a relatively regular fluctuation. Moreover, the flow pulsation amplitude increases with the pressure difference. The flow non-uniformity coefficient under different pressures is calculated by simulation data and proves that it shows an upward trend with the increase of pressure, which indicates that the outlet pressure is an important factor to cause the irregular change of the internal flow field of gear pump.

**Figure 9:**
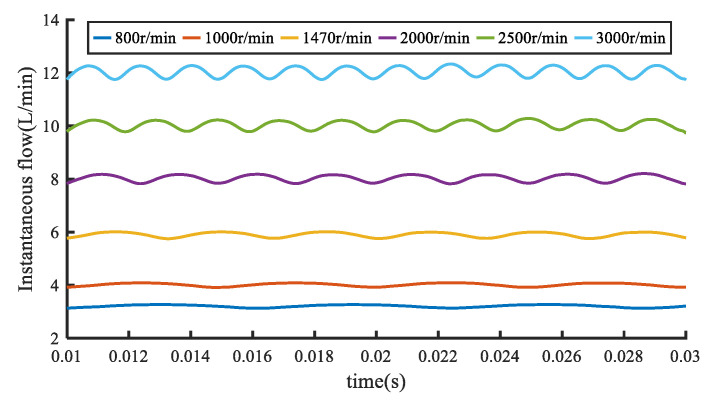
Contrast curves of simulated instantaneous flow of gear pumps at different speeds.

Figure 9 covers the simulated instantaneous flow rate of the gear pump under different speeds. Similar to Figure 8, the flow contrast curve is also read from 0.01 s.

It can be intuitively seen from Figure 9 that both instantaneous flow rate and the flow pulsation rate of the gear pump outlet increase with the speed, which is in accordance with theoretical derivation. After calculating the flow non-uniformity coefficient at different rotating speeds, it is found that the rotating speed which has a positive effect on the convective arterial motility, on the contrary has a negative effect on the flow unevenness coefficient. Therefore, the rotating speed increase can be considered as a method for flow quality improvement. However, the increase of rotating speed will bring some disadvantages such as vibration and noise, so the speed should be selected reasonably [19].

**Figure 10:**
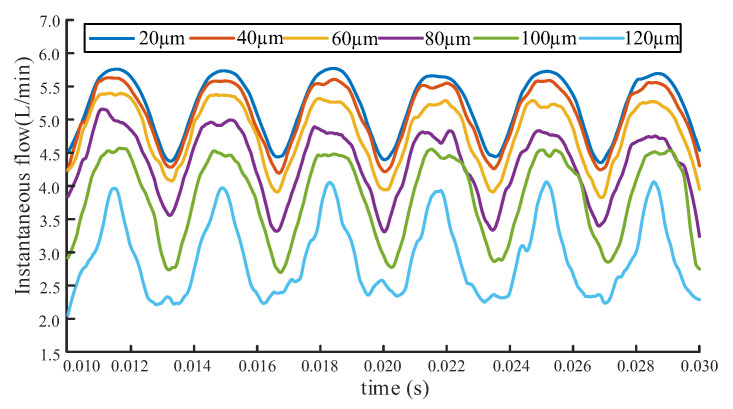
Comparison graph of instantaneous simulation flow rate under different radial wear gaps.

With the rated pressure of 20 MPa and rotating speed of 1470 rpm, the simulation under different radial wear clearance is conducted, and the simulation results are shown in Figure 10.

**Figure 11:**
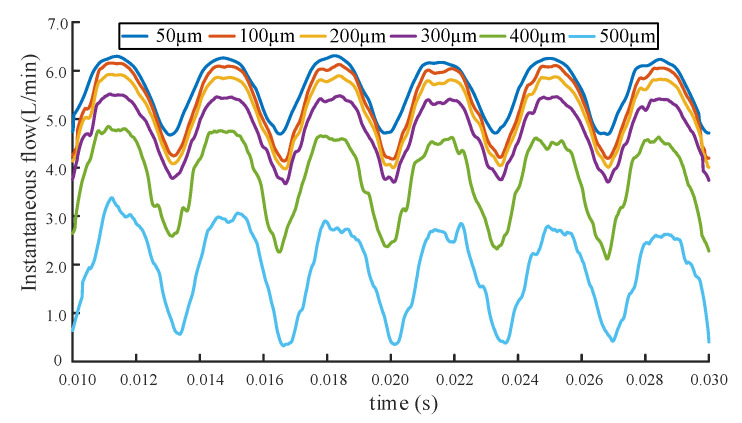
Comparison graph of instantaneous simulation flow rate under different end wear gaps.

It can be clearly observed from Figure 10 that with the increase of radial wear clearance, a significantly decrease of the gear pump flow performance occurred, which is consistent with the expected results. In the final simulation, the end face wear gaps is set to 50 μm, 100 μm, 200 μm, 300 μm, 400 μm, and 500 μm respectively, and they are all selected by reference. Then the simulation under different end wear gaps are processed by using identical parameters as the radial wear gap simulation analysis. The instantaneous simulation flow under different end wear gaps are shown in Figure 11.

It can be seen from Figure 11 that as the wear gap of the end face increases, the instantaneous flow rate gradually decreases, and the flow rate degradation phenomenon becomes more serious. This phenomenon shows that the gear pump has obvious wear degradation.

### 3.4. Degradation Analysis of Simulated Pressure Signal

**Figure 12:**
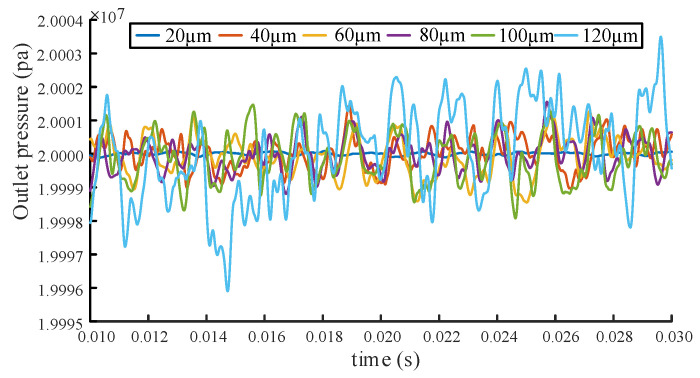
Pressure pulsation curve under different radial wear gaps.

In this part, monitored pressure signals is sorted out as Figure 12 to show the pressure pulsation under different radial wear gaps. It can be seen that all curves fluctuate around the rated pressure with a certain periodicity. The fluctuation amplitude of pressure pulsation increases with the increase of the radial wear gap. It can be considered that with the increase of the radial wear gap, the performance degradation of the gear pump becomes more and more serious [20].

**Figure 13:**
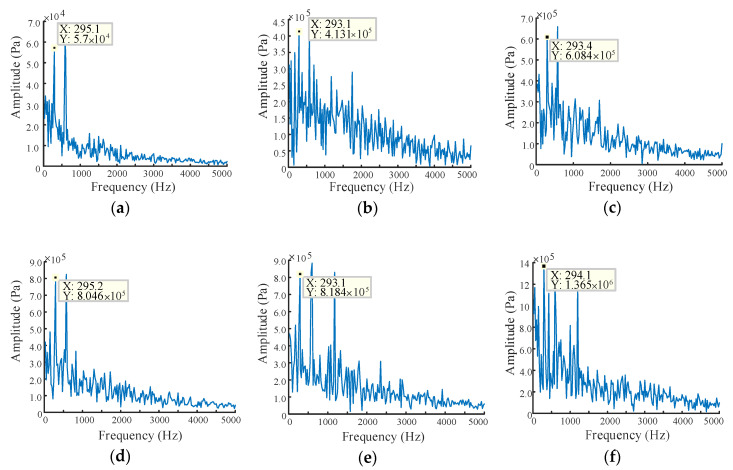
Fast Fourier transform (FFT) of pressure signal under different radial wear gaps: (**a**) wear gap is 20 μm; (**b**) wear gap is 40 μm; (**c**) wear gap is 60 μm; (**d**) wear gap is 80 μm; (**e**) wear gap is 100 μm; (**f**) wear gap is 120 μm.

The pressure signals under different radial wear gaps are subjected to fast Fourier transform (FFT), and the transformation results are shown in Figure 13. The gear pump rotation frequency is 24.5 Hz, the number of teeth is 12, and the tooth frequency is calculated to be 294 Hz in this simulation. As suggested in Figure 13, more frequency components appear with the wear gap increases, which indicates a serious turbulence in the internal flow field of the gear pump [21]. Furthermore, the amplitude increases with the gap, which also fully proves that the performance of the gear pump decreases notably.

**Figure 14:**
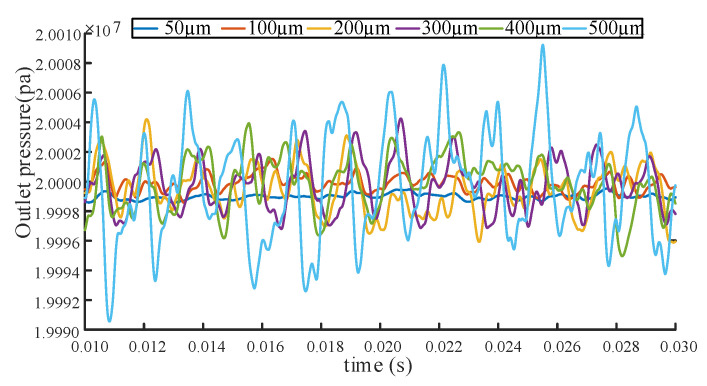
Pressure fluctuation curve under different end wear clearance.

The pressure fluctuation signals under different end wear clearances are concluded in Figure 14.

It can be seen from Figure 14 that each curve fluctuates around the rated pressure with a certain periodicity. Meanwhile, it is also found that the fluctuation tendency of the pressure pulsation increases with wear clearance of the end face, which means a substantial degradation of the gear pump performance [22].

**Figure 15:**
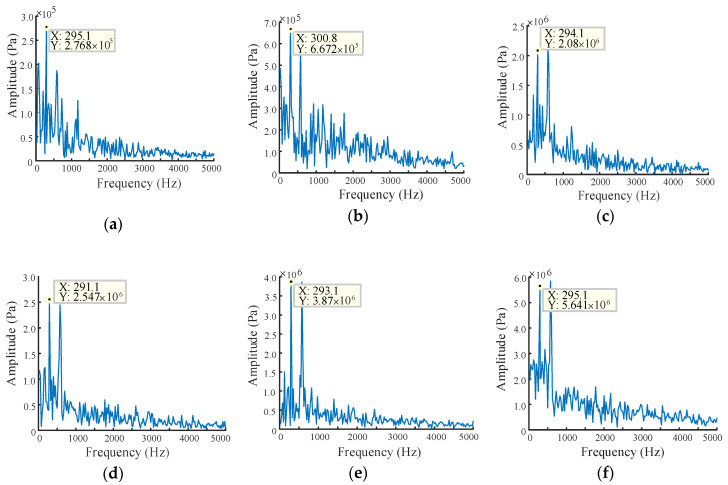
FFT of pressure signal under different end face wear gaps: (**a**) wear gap is 50 μm; (**b**) wear gap is 100 μm; (**c**) wear gap is 200 μm; (**d**) wear gap is 300 μm; (**e**) wear gap is 400 μm; (**f**) wear gap is 500 μm.

The pressure signals under different end face wear gaps are subjected to FFT as reported in Figure 15. It can be demonstrated from the frequency domain distribution of pressure pulsation in Figure 15 that as the wear gap increases, more frequency components appeared, indicating that the turbulence of the flow field becomes more serious. Apart from this, the amplitude are also detected gradually increasing with wear clearance, which fully dedicates that the gear pump has deteriorated in performance.

### 3.5. The Mapping Relationship between Simulation and Theory

**Figure 16:**
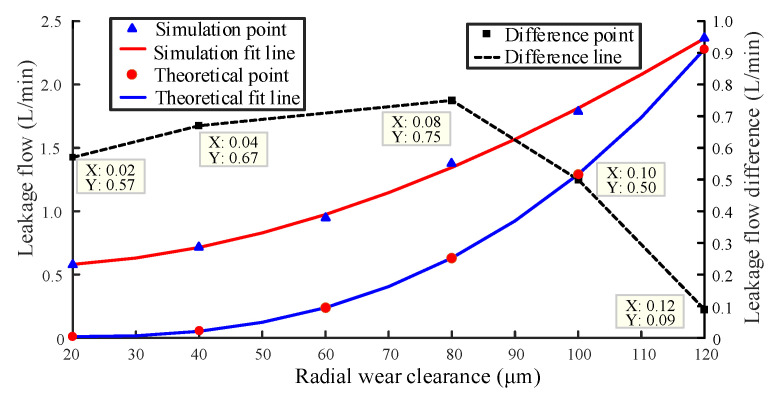
Fitting line of radial wear clearance and leakage.

Cubic function fitting is one of the least square fitting methods with significant fitting effect. The set wear gap is fitted with the leakage data obtained by simulation and theoretical calculation, respectively with the method of cubic function fitting. In Figure 16, the fitting curve of radial wear gap and leakage are compared to evaluate the feasibility of the fitting line obtained by simulation to replace the wear degradation trajectory [23,24].

It can be seen from Figure 16 that the fitting of the two curves is excellent and has a consistent trend. The minimum difference between the simulated leakage flow fitting curve and the theoretical leakage flow fitting curve is 0.09 L/min, and the maximum difference is 0.75 L/min. There is no significant gap between simulation results and theoretical results, which demonstrates a high credibility of the simulation results.

**Figure 17:**
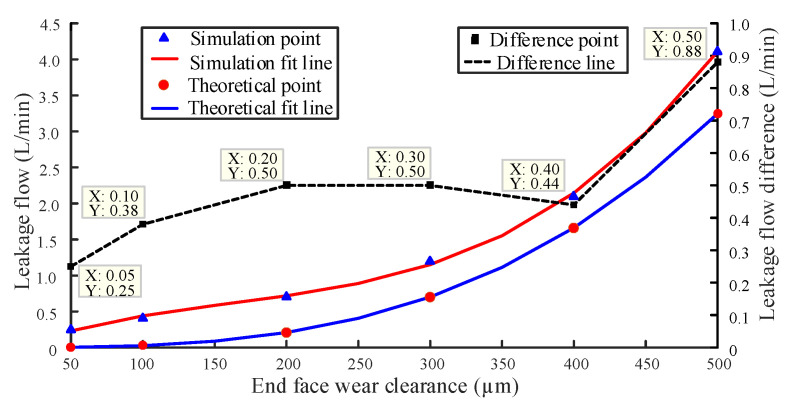
Fitting line of end face wear gap and leakage.

The fitting curve of cubic function of end face wear gap and leakage is shown in Figure 17. As can be seen from the figure, the minimum and maximum differences between the two curves are 0.25 L/min and 0.88 L/min, respectively. Although there are some differences between the two fitting curves, they still have high accuracy.

It can be seen from Figure 16 and Figure 17 that the simulation results and the theoretical calculation results have a better fitting effect and have the same change trend. That is to say, the wear degradation trajectory can be replaced by the simulation curve, both in radial and end face.

## 4. Experimental Validation of the Model

**Figure 18:**
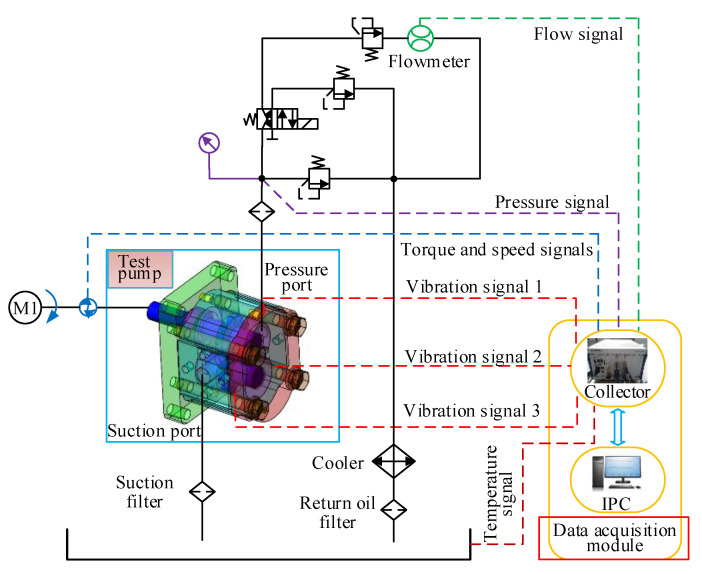
Schematic diagram of the test system.

The principle of the hydraulic system for an accelerated life test (ALT) is shown in Figure 18. Because of the repeated hydraulic circuit, the entire hydraulic system is simplified into a single pump hydraulic circuit in Figure 18. A direct acting overflow valve is selected as a loading valve to set the pressure of the acceleration circuit and the collection circuit. The two-position four-way electromagnetic valve, cooperating with the direct acting overflow valve, is used for switching between the two circuits [25].

**Figure 19:**
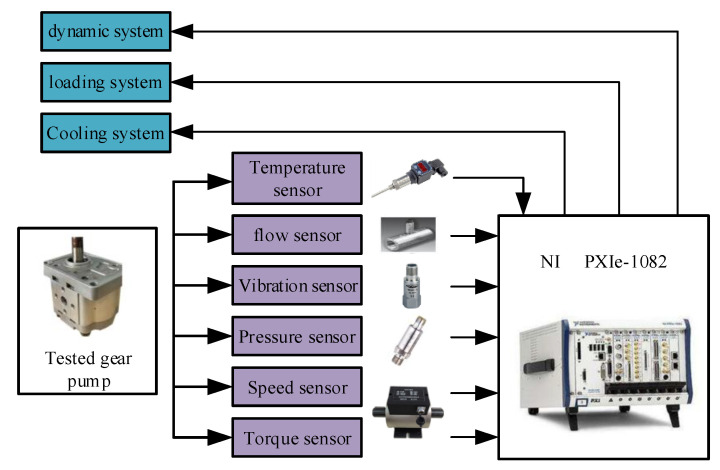
Measurement and control hardware structure of test device.

The test-bed is equipped with temperature sensor to monitor the temperature in real time to ensure the single variable. It is also equipped with pressure and flow sensors to collect pressure and flow data. Acceleration sensors are installed in the vertical direction, horizontal direction and axial direction of the gear pump to measure the vibration of the pump casing. A torque sensor is set at the connection of gear pump and motor to monitor the change of torque and speed. In this experiment, pxie-1082 processor is selected and used together with LabVIEW control panel. The processor is efficient and fast, and integrates signal acquisition and control. The measurement and control hardware structure of the gear pump ALT device is shown in Figure 19.

The sensor parameters used in the experiment are as follows:

- Acceleration sensor: model is YD-36D, sensitivity is 0.002 V/ms^-2^, frequency range is 1~12000 Hz, measuring range is 0~2500 m/s^2^.
- Pressure sensor: model is PU5400, working voltage is 16~30 VDC (Voltage Direct Current), analog voltage output is 0~10 V, measuring range is 0~400 bar.
- Torque speed sensor: model is CYT-302, torque range is 0~20 Nm, torque output is 0~5 V, speed input is 0–3000 rpm, speed output is 0~5 V.
- Temperature sensor: model is CWDZ11, measuring range is −50 °C~+100 °C, supply voltage is 12~36 VDC, output signal is 4~20 mA.
- Flowmeter: model is MG015, nominal diameter 110 mm, flow range is 1–40 L/min, temperature range is −20 °C~+120 °C.

In this experiment, the most widely used gear pump in China is selected, and its model is CBW-F304-CFP. The overflow valve is used as the pressure loading valve to adjust the pressure. According to the structural characteristics of the tested gear pump, the pressure stress is determined as the acceleration stress of this test. On the basis of not changing the failure mechanism, combined with the characteristics of the step-accelerated stress test, three stress levels are selected between the rated pressure of 20 MPa and 30 MPa, they are 23 Mpa, 25 MPa and 27 Mpa respectively. According to the industry standard Hydraulic gear pump JBT7014.2-2018, under the rated working condition, the volume efficiency is less than 82%, which is considered as failure [26,27]. Using a quantitative truncation method, when one of the four pumps reaches the specified amount of degradation for two consecutive measurements, the stress is raised to the next stress stage. According to the principle of single variable, ensure that the pollution degree of hydraulic oil does not exceed NAS 6, the rotation speed is maintained at about 1470 rpm, and the oil temperature is constant at about 50 °C. The test procedure is as follows:

- Disassemble and survey the four gear pumps under test to ensure no wear inside. After disassembly and observation, clean the parts, restore the pump to its original state and install it on the test stand.
- After all test parts are installed, start the machine for pre-test, observe whether the readings of each sensor are normal, ensure the correct rotation direction of the motor, and keep the rotation speed at about 1470 rpm.
- Start the test and record the data. Adjust the loading stress of acceleration circuit to 23 Mpa, and the pressure of data acquisition circuit to 20 MPa. During the experiment, the system has been working under the accelerated stress. Every 10 min, the system will automatically switch to the acquisition branch for data acquisition.
- This experiment uses a quantitative truncation method. When the flow of the external gear pump drops to the specified degradation amount, the stress is increased to the next stage. In the final stress stage, when the flow reaches the specified degradation amount, the test is terminated.

**Figure 20:**
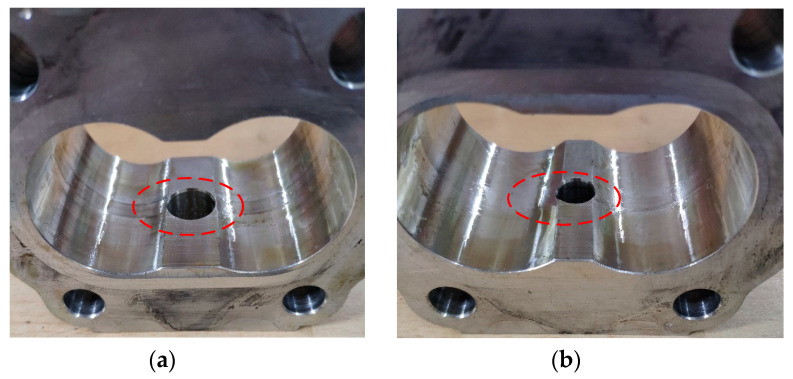
Gear pump radial wear: (**a**) suction port; (**b**) pressure port.

**Figure 21:**
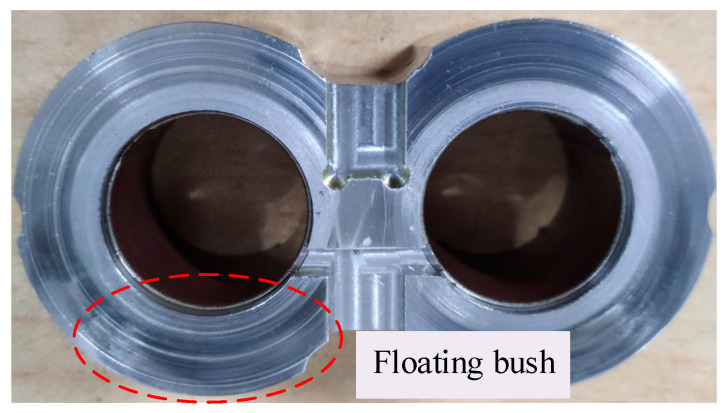
Gear pump end face wear.

The actual wear in radial and end face of the gear pump is shown in Figure 20 and Figure 21.

As observed in Figure 20, the groove of the oil suction chamber is deeper because of the deflection deformation caused by the high pressure of the pressure oil chamber. At the same time, iron chips generated in wear also contributes to the scratches in the pressure oil chamber, which is consistent with the theoretical analysis results. It can be found from Figure 21 that there are many slight scratches on the surface of the floating bush, which is similar to the results of theoretical analysis. This shows that the wear of the end face has a serious effect on the failure of the gear pump.

**Figure 22:**
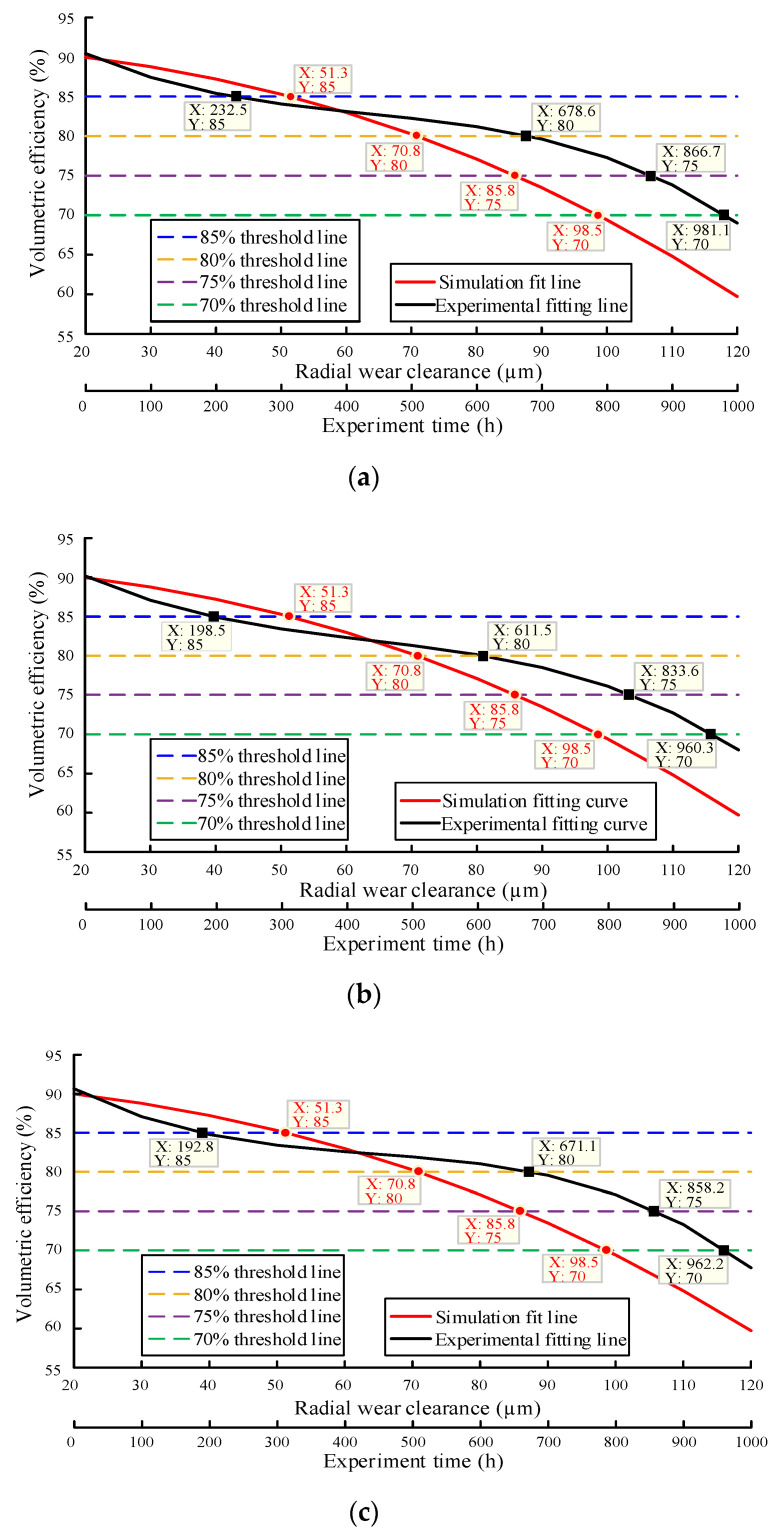

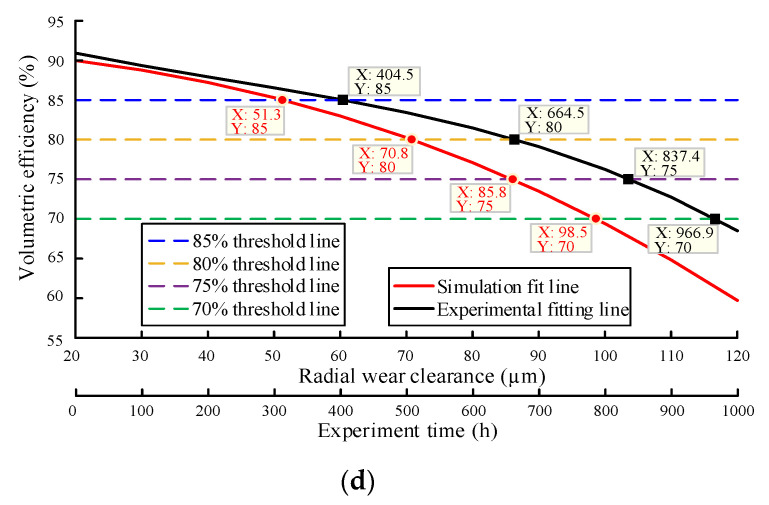
The fitting curve of the test data and the simulated fitting curve under the radial wear clearance: (**a**) Pump 1; (**b**) Pump 2; (**c**) Pump 3; (**d**) Pump 4.

**Figure 23:**
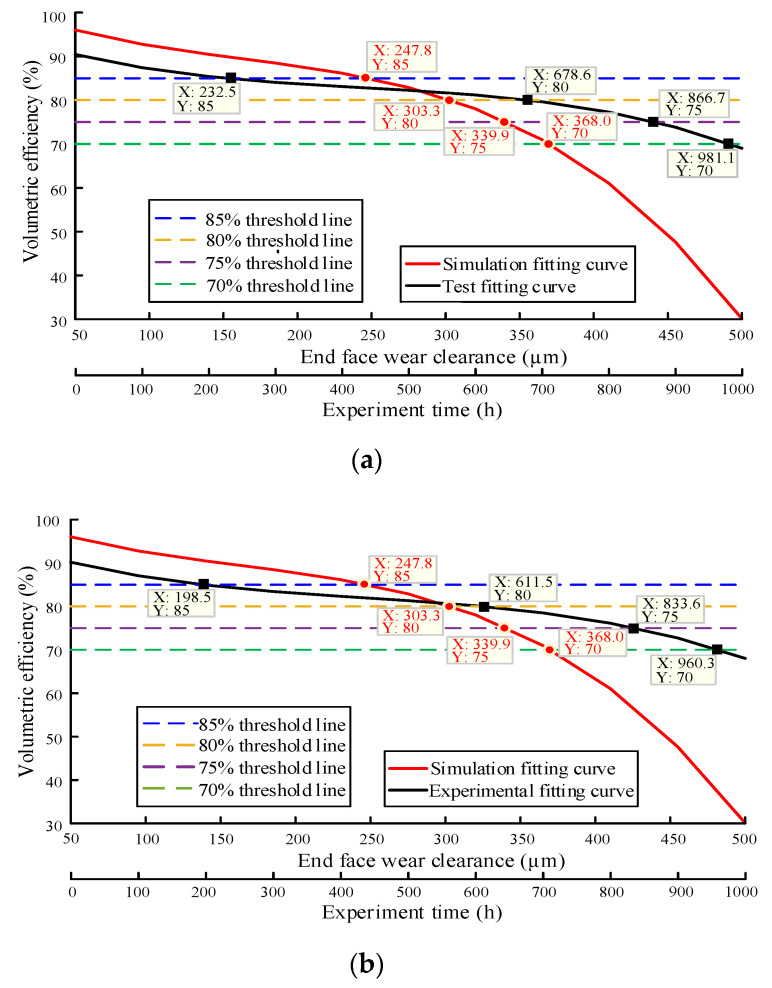

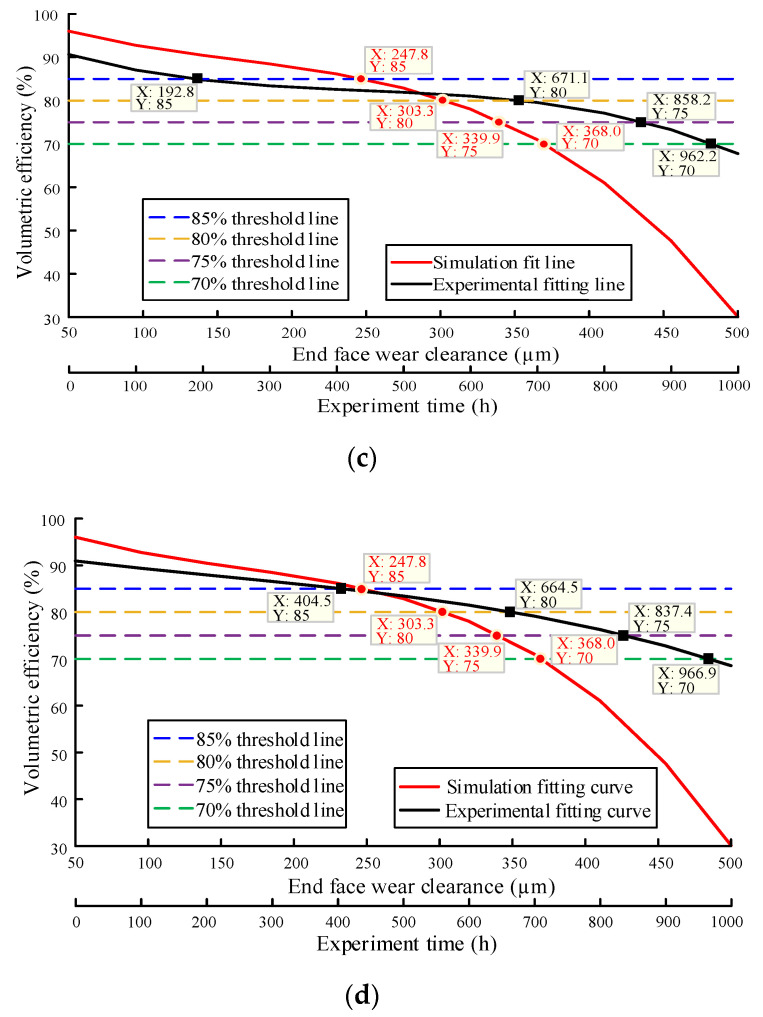
The fitting curve of the test data and the simulated fitting curve under the end face wear clearance: (**a**) Pump 1; (**b**) Pump 2; (**c**) Pump 3; (**d**) Pump 4.

In order to judge the degradation state of the gear pump through the method of flow field simulation calculation, the volumetric efficiency data obtained from the simulation and the volumetric efficiency data obtained from the wear degradation test were fitted with cubic functions. The simulation results are analyzed from two aspects of different radial wear conditions and different end wear conditions, and it is considered that the intersection point between the fitting curve and the specified threshold line is the real degradation point [28,29,30]. The fitting curve of the test data and the simulated fitting curve under the radial wear clearance are shown in Figure 22, and the fitting curve of the test data and the simulated fitting curve under the end face wear clearance are shown in Figure 23.

In Figure 22 and Figure 23, the abscissa corresponding to the simulated fitting curve is the wear gap, and the abscissa corresponding to the experimental fitting curve is the experimental time [31,32]. The reasons are as follows: (i) the relationship between flow rate and time cannot be measured in the simulation, but the change of wear gap with flow rate can be obtained; (ii) the flow rate can be monitored in real time during the experiment, but the change in the degree of wear of the gear pump cannot be monitored in real time [33,34].

It can be seen from the simulation fitting curve that the volumetric efficiency of the four sample pumps decreases with the increase of wear clearance. It can be seen from the fitting curve of the experiment that the volumetric efficiency of the four pumps is in the range of 60–70% after working for 1000 h, all of which are in the state of moderate degradation. The specific degradation of the four sample pumps is summarized in Table 2 and Table 3:

Acquired from Table 2 and Table 3, the working time when the volumetric efficiency of the four pumps drops to 70 % is 981.8 h, 960.3 h, 962.2 h and 966.9 h respectively, and the corresponding radial wear clearance and end wear clearance are 98.5 μm and 368.0 μm, respectively. The wear degradation state of the four sample pumps is effectively predicted.

There are some errors between the simulation results and the test results, and the reasons for these differences are as follows:

- The simplified model is used in the simulation, which is different from the actual model, so there is error.
- The radial wear of gear pump is uneven, and the wear mainly occurs near the oil suction port. However, it is difficult to get the accurate wear value due to the difficulty in measuring the wear degree. In this paper, the radial wear is determined as uniform annular wear in the simulation, so the simulation results will have certain deviation, but it is still of great significance.
- The manufacturer will set a fixed end face clearance for a certain type of pump when producing the gear pump. Due to the existence of floating shaft sleeve, the oil film in the clearance will change constantly, so the end face clearance is always changing in practice. However, in the simulation analysis of end face wear, the end clearance is set as a constant value to deal with, so there will be some errors in the simulation results.

In order to obtain the radial wear and end face wear of the gear pump after wear degradation test, four sample pumps were disassembled, cleaned and dried after ALT of the gear pumps, and the wear was measured by profile measuring instrument. The measuring positions are respectively the inner side of the gear pump housing and the end face of the floating shaft sleeve, corresponding to the radial wear and the end face wear, respectively.

**Figure 24:**
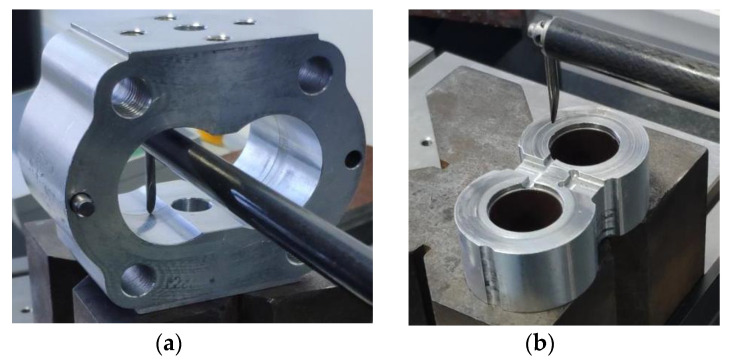
Enlarged drawing of wear measurement part: (**a**) radial wear measurement; (**b**) end face wear measurement.

The German MarSurf XC20 profilometer is selected as the experimental equipment. The profilometer can perform the measurement requirements of different categories of complex profiles. Figure 24 is an enlarged view of the wear measurement area.

The measurement results of wear show that the radial wear gap is about 70 μm on average, and the end face wear gap is about 20 μm on average. When the radial clearance and the end face clearance are set to 20 μm and 50 μm, the radial and end wear values obtained through simulation are correspondingly 90 μm and 70 μm. This indicates that the radial wear value is closer to the experimental results, and proves that compared with the end face wear, radial wear is the main cause of the volumetric efficiency reduction in the wear degradation test of the gear pumps, which is consistent with the actual situation.

## 5. Conclusions

This paper presents a method for identifying the degradation state of gear pump wear based on flow-field analysis, and verifies the accuracy of the method through theoretical calculations. A gear pump stepping stress accelerated wear degradation test bench was established, and the wear degradation data of the sample pump was obtained and compared with the simulation results. Finally, combined with the cubic function fitting method, the wear and tear degradation state of the sample pump was effectively identified. The following conclusions have been drawn:

1. The simulation results show that the instantaneous flow rate of the gear pump decreases with the increase of the pressure difference, while the fluctuation amplitude and non-uniformity coefficient of the flow increase with the increase of the pressure difference. It is proved that the increase of the outlet pressure is an important factor causing the turbulence of internal flow field of the gear pump, and indirectly proves that increasing the pressure will accelerate the internal wear of the gear pump.
2. The simulation results show that the instantaneous flow and flow pulsation rate of the gear pump increase with the increase of the speed, but the flow non-uniformity coefficient decreases with the increase of the speed. It is shown that increasing the rotating speed can be used as a method to obtain the steady flow rate.
3. The simulation results show that with the increase of wear clearance, the instantaneous flow rate of the gear pump gradually decreases, and the performance degradation characteristics are very obvious. It can be seen that the simulation results are highly consistent with the theoretical and experimental results. In addition, through the comparative analysis of the simulation results and the real wear, it is confirmed that the radial wear is the main reason for the wear degradation of the gear pump.

End face wear and radial wear usually occur together. The text only analyzes it separately. In future work, both types of wear will be studied together.

## Figures and Tables

**Table 1:** Model verification parameter comparison table.

Model Category	Theoretical Flow	Simulation Flow	Flow Difference
2D model	5.88 L/min	5.86 L/min	0.02 L/min
3D model	5.88 L/min	5.85 L/min	0.03 L/min

**Table 2:** Degradation state of wear clearance of sample pump.

Volumetric Efficiency	Radial Wear Clearance	End Face Wear Clearance
85%	51.3 μm	247.8 μm
80%	70.8 μm	303.3 μm
75%	85.8 μm	339.9 μm
70%	98.5 μm	368.0 μm

**Table 3:** Time for sample pump to reach each degradation stage.

Volumetric Efficiency	Pump 1 Time	Pump 2 Time	Pump 3 Time	Pump 4 Time
85%	232.5 h	198.5 h	192.8 h	404.5 h
80%	678.6 h	611.5 h	671.1 h	664.5 h
75%	866.7 h	833.6 h	858.2 h	837.4 h
70%	981.8 h	960.3 h	962.2 h	966.9 h
